# Blood Pressure and Hypertension Among Adults Aged 80 and Above: Findings From the Population‐Based German Health Survey Gesundheit 65+

**DOI:** 10.1155/ijhy/2366213

**Published:** 2026-07-09

**Authors:** Dorothea Möẞnang, Julia Charlotte Büschges, Giselle Sarganas, Beate Gaertner, Judith Fuchs, Hannelore Neuhauser

**Affiliations:** ^1^ Department of Epidemiology and Health Monitoring, Robert Koch Institute, Berlin, Germany, rki.de; ^2^ DZHK (German Centre for Cardiovascular Research), Berlin, Germany, dzhk.de

**Keywords:** aged, aged 80 and over, awareness, control, hypertension^∗^/epidemiology, hypertension^∗^/therapy, treatment

## Abstract

**Background:**

Hypertension is highly prevalent in older adults; therefore, hypertension management remains essential. Nevertheless, population data is scarce. Until recently, population blood pressure (BP) monitoring in Germany only included the population up to age 79. This study investigates BP and hypertension management in octogenarians and nonagenarians in Germany from the study Gesundheit 65+.

**Methods:**

In 2022–2023, brachial BP measurements (Mobil‐O‐Graph, IEM GmbH, Aachen/Germany) and health questionnaires were conducted in 807 participants aged 80–99 years of the German population‐based Gesundheit 65+ study. Hypertension was defined as BP ≥ 140/90 mmHg, or antihypertensive treatment in participants with self‐reported hypertension. Hypertension control was defined as BP < 140/90 mmHg among participants with hypertension. Weighted prevalence of hypertension, awareness, treatment and control by sex were estimated with 95% confidence intervals (CIs). Partitioning Around Medoids cluster analysis was performed to identify distinct subtypes among participants with hypertension.

**Results:**

The prevalence of hypertension in women was 77.8% [95% CI: 71.4–83.2] and in men 72.3% [65.7–78.0]. Among those with hypertension, 85.2% were aware, 81.7% treated, and 57.3% controlled. Control was significantly lower in women compared to men, with half of women having controlled hypertension, compared to two‐thirds of men. The cluster analysis revealed two subgroups among women with hypertension: a well‐treated/controlled but multimorbid group and an untreated/uncontrolled but subjectively healthier group. Three clusters emerged among men with hypertension: treated/controlled, less treated/uncontrolled and a severely multimorbid cluster with high treatment and control rates.

**Conclusion:**

Among octogenarians and nonagenarians in Germany, hypertension prevalence is higher in women than in men, while hypertension control among women is lower. This underscores the need for more attention to BP in older women. Furthermore, our findings indicate that high BP, as a largely asymptomatic health risk, may be inadequately controlled among otherwise healthy older adults.

## 1. Introduction

The WHO’s 2025 global report on hypertension emphasises that hypertension is one of the leading modifiable risk factors for cardiovascular morbidity and mortality, making it an urgent global public health threat [1]. Its incidence and prevalence steeply increase with age [2], and the proportion of disability‐adjusted life years caused by hypertension increases from 6% in the 25–49 age group to 20% in the over‐75 age group [3].

The risk of adverse events associated with hypertension, such as stroke and heart attack, can still be reduced in older age with antihypertensive treatment [4, 5]. Therefore, many national and international guidelines recommend antihypertensive therapy for people aged over 80 [6]. However, there is also evidence of adverse events associated with lower blood pressure (BP) levels under antihypertensive treatment, e.g., cognitive decline and higher all‐cause mortality [7, 8]. These findings highlight the challenges of treating old and very old adults with hypertension [9, 10]. To address these challenges, a differentiated patient‐centred approach that considers the benefits and risks of pharmacological treatment for frail and very old patients is recommended [11].

Despite growing awareness of high BP in old and very old adults, population‐wide data on the prevalence, awareness, treatment and control of hypertension in people over 80 is scarce. Most large‐scale epidemiological studies in Germany limited their research to individuals up to 79 years of age [12–14]. The few studies examining BP in people above 80 were conducted more than a decade ago [15–17]; an update of these results is therefore needed. The Study on Health of Older People in Germany (Gesundheit 65+) provides population‐based data on the health of people aged 65 and over in Germany [18].

Old adults form a very heterogeneous group ranging from healthy individuals to patients with a high disease burden [19]. Thus, a better understanding of this heterogeneity could lead to more effective treatment and care [20]. This work may provide new insights into hypertension in people aged 80 and over, thereby contributing to improved BP care and management in older age. This study aims to (1) investigate the prevalence, awareness, treatment and control of hypertension in people aged 80 years and older in Germany, as well as the distribution of systolic BP (SBP) and diastolic BP (DBP) and (2) to explore different phenotypes of very old adults with hypertension that can be identified by clustering of clinical and sociodemographic variables in order to inform hypertension management.

## 2. Methods

### 2.1. Study Population

Data from standardised BP measurements of the old and very old population in Germany from the nationwide population‐based study Gesundheit 65+ were used [18]. The target population of the study was German‐speaking individuals 65 years and older permanently living in Germany. The study was conducted from June 2021 to April 2023 using a two‐stage random sampling process via population registries and comprised a baseline questionnaire as well as three follow‐up questionnaires administered within a one‐year period. Individuals incapable of completing the questionnaires themselves had the option of appointing a proxy. 2244 individuals (1082 women and 1162 men) who were 79 years and older participated in the baseline survey of Gesundheit 65+ (baseline total response rate: 28.2%). At the last follow‐up, when they were one year older compared to baseline, a subsample of participants from each sampling point was examined in their private households or nursing homes (408 women and 399 men aged 80 years and older, reparticipation rate: 35.9%).

The study was primarily funded by the Federal Ministry of Health, Germany (Grant No: ZMVI1‐2518FSB410), and approved by the ethics committee at the Berlin Chamber of Physicians (German: Berliner Ärztekammer, Eth‐50/19) and the data protection officer of the Robert Koch Institute (RKI). It was conducted in compliance with the EU General Data Protection Regulation and the Federal Data Protection Act. Participants or their legal representatives provided written informed consent prior to examination and at the time of baseline participation. Comprehensive information on the Gesundheit 65+ study has been previously published [18, 21].

### 2.2. Measurements and Definitions

BP was measured during the examination one year after baseline on the upper arm using an automated oscillometric device with appropriately sized upper arm cuffs (Mobil‐O‐Graph, IEM GmbH, Aachen, Germany). After a five‐minute rest period, three BP measurements were taken at two‐minute intervals while the participants sat with their back supported, arm resting on a table, legs uncrossed and feet flat on the floor. Extreme BP values were examined individually, considering all measurements obtained. Values classified as erroneous or not measured according to the protocol were excluded from further analysis. At least one paired SBP/DBP value was required from the second or third BP measurement for it to be considered valid. SBP and DBP were calculated as the mean of the second and third measurements.

Applied definitions were as follows: (1) Hypotensive BP: SBP < 90 and/or DBP < 50 mmHg. (2) Normotensive BP: SBP 90–139 and DBP 50–89 mmHg. (3) Hypertensive BP: SBP ≥ 140 and/or DBP ≥ 90 mmHg. (4) Isolated systolic hypertension (ISH): SBP ≥ 140 and DBP < 90 mmHg. (5) Hypertension: hypertensive BP and/or self‐reported hypertension and treatment as defined below. (6) Awareness: participants with hypertension who reported a hypertension diagnosis. (7) Treatment: intake of medication with antihypertensive main effect according to Anatomical Therapeutic Chemical Classification System (ATC) including diuretics, beta‐blockers, calcium channel blockers, ACE inhibitors, angiotensin receptor blockers and other antihypertensive drugs (ATC codes: C02, C03, C07, C08, C09) in the seven days prior to examination. As the indication for these medications may be other than hypertension, this information was only used if participants were aware of hypertension. This definition is based on previous work [22]. Monotherapy refers to treatment with antihypertensives from a single active ingredient. Polytherapy was defined as the use of antihypertensive medication with several antihypertensive agents [23]. (8) Control: SBP < 140 and DBP < 90 mmHg among participants with treated hypertension. An alternate threshold for controlled hypertension was calculated (SBP ≤ 150 mmHg), which is used for people over 80 in various guidelines [6].

### 2.3. Covariates

Covariates were either objectively measured during the examination or self‐reported at the final follow‐up questionnaire for further information relating to physical and mental health. Sociodemographic information was assessed at baseline.

The following variables were used to describe physical health: Body height and weight were measured using a portable stadiometer (Seca 213, Seca, Hamburg, Germany) and a body weight scale (Seca 208, Seca, Hamburg, Germany). Body mass index (BMI) is weight in kilograms divided by height in meters squared (kg/m^2^). Participants with a BMI ≥ 30 kg/m^2^ were categorised as obese [24]. Subjective health was assessed using the question, ‘How is your health in general?’, categories ‘good’ and ‘very good’ were compared to ‘fair’, ‘bad’ and ‘very bad’. Multimorbidity was defined as the presence of two or more diseases and health problems out of a list of 11 chronic diseases (incl. cardiovascular diagnoses) [25]. Frailty components according to Fried and colleagues [26] were analysed: self‐reported low energy, unintentional self‐reported weight loss, low self‐reported physical activity, self‐reported walking difficulties and low measured grip strength.

The following variables were used to describe mental health: Depressive symptoms experienced over the past two weeks were evaluated using the two‐question Patient Health Questionnaire (PHQ‐2) [27]. A total score of three or more was considered indicative of depressive symptoms. General life satisfaction was assessed using the question, ‘How satisfied are you overall with your current life?’, according to Richter et al. [28]. A score of seven or more was considered an indication of high or very high life satisfaction [29]. Subjective memory impairment was evident if participants reported a decline in their memory with associated worries [30].

The following variables were used to describe sociodemographic characteristics: Information on sex, month and year of birth was obtained from local population registers at the time of sampling. Long‐term care level (short: care level) that entitles to long‐term care benefits under the German statutory long‐term care insurance system was recorded using a yes/no question. The educational group was categorised based on the Comparative Analyses of Social Mobility in Industrial Nations (CASMIN) [31]. Housing at the time of the final follow‐up was categorised into private households, nursing homes or special housing for older people.

### 2.4. Statistical Analysis

Descriptive analyses used an available‐case approach and were conducted sex‐stratified. Continuous variables were presented as means; categorical data were expressed as prevalence or relative frequencies. Differences were evaluated using a design‐based *t*‐test. To account for the complex survey design and obtain population‐representative estimates, all analyses were conducted using survey procedures and a weighting factor [18]. The weights adjusted the sample to match the German population aged 80+ as of 31 December 2020. They incorporated sex, age, region, municipality size, educational attainment and variables that accounted for the loss to follow‐up [18]. Estimates were reported with 95% confidence intervals (CIs); *p* < 0.05 was defined as significant.

We conducted an exploratory cluster analysis to identify subgroups of patients with hypertension and followed the practical *Qluster* workflow, which is designed for robust, easy‐to‐implement clustering of mixed health data [32]. The variables provided for the cluster analysis covered a mixed dataset of sociodemographic and health‐related topics, including age, sex, cardiovascular diagnoses, BP measurements and anthropometric measurements, as well as self‐reported information on physical and mental health from the Gesundheit 65+ data. Therefore, only the subsample of people over the age of 80 with hypertension, as defined in our study, was included in the cluster analysis. Missing values were handled using multiple imputation by chained equations. As sex differences in hypertension were evident, the following steps were carried out separately for each sex: factor analysis of mixed data (FAMD) to reduce the dimensions of mixed variables and Partitioning Around Medoids (PAM) clustering [33, 34]. Cluster stability was assessed using Jaccard similarity. Finally, we described and compared the resulting clusters a posteriori by means and frequencies. A more detailed description of the handling of missing data and imputation models, as well as a detailed explanation of the PAM clustering process, is provided in the Supporting A. All analyses were conducted in R (Version 4.4.1; R Core Team, 2023) and in RStudio (Version 2024.04.0; Posit Software, PBC). The following packages were used: *tidyverse* [35], *survey* [36], *mice* [37], *cluster* [34] and *factoextra* [38].

## 3. Results

### 3.1. Sample Description

Of the 807 participants aged 80 years and older, 96.7% (*n* = 781) had valid BP measurements as well as complete information on hypertension diagnosis and/or antihypertensive medication and were included in the analysis. A total of 50.5% of participants were women (49.5% men). On average, the participants were 84.9 years old, and the maximum age was 99 years. Two‐thirds reported multimorbidity; in more than one‐third of the sample, the frailty components low physical activity, walking difficulties and low grip strength were present. Most of the sample were living in their private household; a mere 5% of participants resided in nursing homes (Table 1).

**TABLE 1:** Key characteristics of the study population aged 80 years and older from the nationwide examination survey Gesundheit 65+, 2022‐2023.

	Women (*n* = 394)		Men (*n* = 387)		Total (*n* = 781)	
	%	[95% CI]	%	[95% CI]	%	[95% CI]
Age (mean in years)	85.1	[84.4–85.8]	84.6	[84.1–85.2]	84.9	[84.4–85.4]
Age groups						
80–84 years	51.7	[44.6–58.9]	56.8	[49.6–63.9]	53.8	[48.6–59.0]
85–89 years	32.2	[25.8–38.6]	31.2	[24.1–38.3]	31.8	[27.0–36.7]
90+ years	16.0	[9.8–22.2]	12.0	[6.8–17.2]	14.4	[10.2–18.6]
Educational group						
Low	70.1	[64.3–75.9]	62.7	[56.1–69.3]	67.1	[62.9–71.2]
Medium	24.4	[18.7–30.0]	21.8	[15.9–27.7]	23.2	[19.4–27.3]
High	5.5	[3.6–7.5]	15.5	[11.9–19.1]	9.6	[7.7–11.5]
Housing						
Private household	94.6	[91.0–98.3]	95.7	[92.5–98.9]	95.1	[92.5–97.6]
Nursing home/special housing	5.4	[1.7–9.0]	4.3	[1.1–7.5]	4.9	[2.4–7.5]
Physical health						
Obesity	28.8	[22.1–36.5]	18.4	[13.6–24.4]	24.6	[20.0–29.8]
(Very) Good subjective health	31.4	[25.2–37.6]	44.4	[36.9–51.8]	36.6	[31.8–41.5]
Multimorbidity^1^	73.0	[78.2–88.4]	70.3	[68.6–80.8]	71.8	[66.9–76.8]
Frailty components						
Low energy	24.4	[18.3–31.6]	18.7	[13.2–25.8]	22.1	[17.8–27.1]
Unintentional weight loss	4.5	[2.5–7.8]	3.5	[1.8–6.9]	4.1	[2.6–6.3]
Low physical activity	41.3	[34.1–48.8]	34.2	[27.4–41.8]	38.5	[33.3–43.9]
Walking difficulties	40.0	[33.8–48.5]	30.1	[22.8–38.5]	36.7	[31.5–42.2]
Low grip strength	41.0	[33.8–48.5]	30.1	[22.8–38.5]	45.7	[40.5–51.0]
Antihypertensive medication						
No antihypertensive medication	19.9	[14.6–25.2]	18.1	[12.4–23.8]	19.2	[15.1–23.0]
Monotherapy	17.8	[12.5–23.1]	20.7	[14.9–26.6]	19.0	[15.2–23.1]
Polytherapy	62.3	[55.6–69.1]	61.1	[53.9–68.3]	61.8	[56.9–66.7]

Abbreviation: CI, confidence interval.

^1^Defined as the presence of two or more diseases and health problems out of the following: hypertension, hyperlipidaemia, diabetes, coronary heart disease, stroke (incl. long‐term complications), chronic bronchitis, arthritis, osteoporosis, low back pain, cancer and depression.

### 3.2. Distribution of BP and Hypertension Prevalence, Awareness, Treatment and Control

The results for BP and hypertension among the whole study population are shown in Table 2. Hypertension prevalence was 75.6% [95% CI: 71.1–79.6] in total, 77.8% in women and 72.3% in men. In more than one‐third of women, BP measurements showed hypertensive SBP or DBP. In men, this was measured in one in five cases, which is significantly lower than among women. ISH was found in one in five women, more than twice as often as in men. Low BP occurred significantly less frequently in the sample: 1.6% of women and 8.2% of men had hypotensive BP values.

**TABLE 2:** Prevalence of hypo‐, normo‐ and hypertensive BP, isolated systolic hypertension, hypertension and proportion of awareness, treatment and control of hypertension in the 80 years and older from the Gesundheit 65+ study in Germany, 2022‐2023.

	Total % [95% CI]	Women % [95% CI]	Men % [95% CI]	*p* value
Blood pressure (all study participants)				
< 100/50 mmHg (hypotensive)	4.3 [2.5–7.3]	1.6 [0.7–3.6]	8.2 [4.3–15.2]	0.015
100–139/50–89 mmHg (normotensive)	63.6 [58.2–68.7]	59.4 [51.8–66.6]	69.8 [62.2–76.5]	0.050
≥ 140/90 mmHg (hypertensive)	32.1 [27.2–37.5]	39.0 [31.8–46.6]	21.9 [16.5–28.5]	< 0.001
≥ 140/< 90 mmHg (isolated systolic hypertensive)	16.1 [12.5–20.4]	21.0 [15.7–27.6]	8.8 [5.7–13.3]	< 0.001
Hypertension prevalence (all study participants)				
Hypertension prevalence	75.6 [71.1–79.6]	77.8 [71.4–83.2]	72.3 [65.7–78.0]	0.204
Awareness and treatment (study participants with hypertension)				
Awareness of hypertension	85.2 [80.2–89.2]	84.3 [76.9–89.6]	86.8 [79.4–91.8]	0.574
Treatment of hypertension	81.7 [76.4–86.0]	81.7 [74.1–87.5]	81.6 [73.5–87.6]	0.981
Treatment (study participants aware of hypertension)				
Treatment of hypertension	95.8 [92.0–97.9]	97.0 [91.9–98.9]	94.0 [84.9–97.8]	0.390
Control of hypertension (study participants with hypertension)				
< 140/90 mmHg	57.3 [50.9–63.4]	50.8 [42.2–59.4]	67.4 [58.9–75.0]	< 0.001
< 150/90 mmHg	77.8 [71.9–82.7]	71.3 [62.8–78.6]	88.2 [82.2–92.4]	< 0.001
Control of hypertension (study participants with treated hypertension)				
< 140/90 mmHg	70.1 [63.1–76.3]	62.2 [52.4–71.0]	82.7 [75.4–88.3]	< 0.001
< 150/90 mmHg	81.6 [75.4–86.5]	75.1 [65.9–82.5]	91.9 [87.0–95.1]	< 0.001

Abbreviation: CI, confidence interval.

Among individuals with hypertension, 85.2% [80.2–89.2] were aware of their hypertension and 81.7% [76.4–86.0] were currently treated with antihypertensives (Table 2). There were no statistically significant differences in awareness or treatment between men and women. Overall, 57.3% [50.9–63.4] of participants with hypertension had controlled BP. Clear sex differences existed here: Among women with hypertension, only half had controlled BP compared to more than two‐thirds of men. When considering only those participants who were aware of their hypertension, more than 9 out of 10 were receiving treatment. Considering only those who were treated, 70.1% [63.1–76.3] had controlled BP. There was also a statistically significant difference in BP control among treated men and women (women: 62.2% vs. men: 82.7%). Allowing an SBP of < 150 mmHg for defining control leads to a 10‐ to 20‐percentage point improvement in the control rate for both sexes.

The distribution of mean SBP and DBP is summarised in Table 3. Mean SBP in the entire study population was 130.5 mmHg, and mean DBP was 77.4 mmHg. Mean SBP differed significantly by sex (*p* < 0.001). In women, mean SBP was 134.6 mmHg, while in men, it was 124.2 mmHg. There was less difference in DBP between men and women. No difference in mean SBP and DBP was found between age groups. The mean SBP and DBP were found to be lower among participants who were aware of their hypertension (*p* < 0.001), which is also seen among participants who were receiving antihypertensive treatment in comparison to those not receiving treatment (*p* < 0.001).

**TABLE 3:** Mean SBP and DBP in adults aged 80 years and older by sex, age, awareness and treatment of hypertension from the Gesundheit 65+ study in Germany 2022–2023.

	SBP in mmHg mean [95% CI]	DBP in mmHg mean [95% CI]
Total	130.5 [128.3–132.8]	77.4 [76.1–78.7]
Sex		
Women	134.6 [127.5–133.0]	78.6 [76.7–80.5]
Men	124.2 [121.6–126.8]	75.6 [73.9–77.2]
Age group in years		
80–84	130.3 [127.5–133.0]	78.6 [77.0–80.2]
85+	130.8 [127.1–134.6]	75.9 [73.8–78.0]
Awareness		
Not aware	151.3 [146.9–155.7]	88.1 [85.0–91.1]
Aware	132.2 [129.3–135.1]	77.7 [76.0–79.3]
Treatment		
Not treated	151.4 [146.6–156.3]	93.9 [85.5–102.3]
Treated	131.5 [128.6–134.5]	77.2 [75.5–78.9]

Abbreviations: CI, confidence interval; DBP, diastolic blood pressure; SBP, systolic blood pressure.

### 3.3. Identified Clusters

**FIGURE 1:**
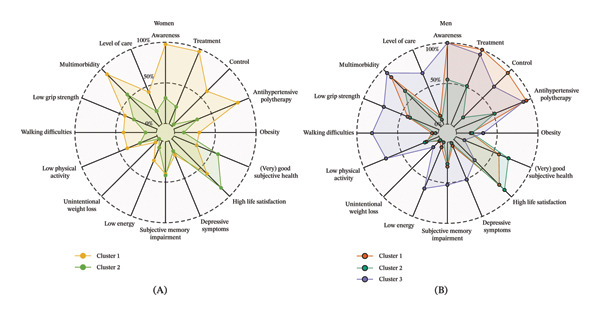
Distribution of sociodemographic, anthropometric and health status characteristics of participants according to clusters identified in women and men, selected according to importance for cluster assignment or for the cardiovascular description of the cluster (A) women; (B) men.

Cluster analysis was conducted to identify subgroups of older adults with hypertension according to BP management and related health factors, without a priori assumptions. A total of 582 individuals with hypertension were available for the final clustering. Among women, two clusters were found (Figure 1A). Cluster 1 (*n* = 241, 77%), the ‘controlled but multimorbid’, and Cluster 2 (*n* = 71, 23%), the ‘uncontrolled, but subjective healthier’, did not differ in age, but Cluster 2 had substantially higher BP. Consistent with this, awareness, treatment and control of BP were very high in Cluster 1, but very low in Cluster 2 (all *p* < 0.001). Use of antihypertensive medication and polytherapy was far more common in Cluster 1, whereas Cluster 2 largely reflected monotherapy or no treatment. Beyond BP management, Cluster 1 showed higher multimorbidity and more frequent frailty components. Obesity was more prevalent in Cluster 1. In contrast, women in Cluster 2 more often reported good subjective health and high life satisfaction, while depressive symptoms did not differ. Overall, the clusters in women represented a treated, controlled but more multimorbid and frail group versus a largely untreated, less multimorbid group with poor BP control.

Among men, three clusters were identified (Figure 1B). Cluster 1 (*n* = 111, 41%), the ‘well‐treated and controlled’, had the lowest BP and a well‐managed profile with near‐complete awareness, treatment and very high control (94%), alongside frequent antihypertensive polytherapy. Cluster 1 is characterised by a high presence of multimorbidity (86%). Cluster 2 (*n* = 96, 36%), the ‘uncontrolled’, reflected a subgroup with markedly elevated BP, lower awareness and treatment and a very low control rate. Subjective health and life satisfaction were highest in this cluster, and multimorbidity was least prevalent. Cluster 3 (*n* = 63, 23%), the ‘vulnerable’, showed moderate BP, high awareness, treatment and high control. Polytherapy was at a very high level in this cluster. It is also distinguished by a pronounced vulnerability burden: frailty indicators, multimorbidity, depressive symptoms and care level were most prevalent. Supporting B provides further summary statistics of the identified clusters, including sociodemographics, physical and mental health and BP management factors.

Across sexes, the dominant cluster in both women and men reflected high awareness and treatment with better BP control and broader medication use, while poorer‐control clusters were characterised by lower awareness and treatment. A key sex difference was the additional male cluster dominated by frailty and depressiveness, whereas in women, multimorbidity was concentrated within the treated/controlled cluster. Cluster stability was moderate to robust: Jaccard means were 0.81/0.68 for female clusters and 0.83/0.77/0.91 for male clusters.

## 4. Discussion

This study presents recent population‐based data on BP and hypertension in the population in Germany from the age of 80. Three out of four people have hypertension, with women (77.8%) being slightly more affected than men (72.3%). Among these numerous older adults with hypertension, awareness (85.2%) and treatment (81.7%) are high and without significant sex differences, but control is lower in women (50.8%) compared to men (67.4%). Using cluster analysis, we examined older adults with hypertension and identified two clusters in women and three in men. These clusters differed in terms of their BP management and other health factors.

Previous evidence on hypertension prevalence and management in adults aged 80 years and older from population surveys is limited. Our findings on hypertension prevalence largely align with those reported previously in Europe [17, 39–41]. There are no Germany‐wide studies, only regional studies. KORA‐Age1, conducted in 2008/2009 in the region of Augsburg, Germany, and including participants aged 65 to 94, found a hypertension prevalence of 73.6% among women and men aged between 85 and 94, which is marginally lower than the results of Gesundheit 65+ [17]. Further evidence has been documented from Poland: In the PolSenior study (2007–2011), a higher prevalence among women aged 80–84 and a similar prevalence among men in the same age group has been observed, compared to our results [41]. More recent results of the NOMED‐AF study (2017–2018) from Poland found that 90% of women and 80% of men over 80 had hypertension, substantially more prevalent than in Gesundheit 65+ [39].

The awareness of hypertension and treatment among individuals aware of their hypertension reported in KORA‐Age1 are similar to ours, exceeding 80% and 95%, respectively [17]. The NOMED‐AF study also reported very similar results regarding awareness and treatment [39]. The PolSenior results from the previous decade were lower than ours, with a treatment rate of around two‐thirds [41].

BP control in KORA‐Age1 was at a similar level among women as in Gesundheit 65+, but lower among men [17]. Our results revealed a significant sex‐specific discrepancy in hypertension control. Earlier studies from Canada and the US have already reported age‐specific sex differences in BP control. In these studies, poorer control was observed in older women and younger men [42, 43]. In the Polish NOMED‐AF study, too, men had better BP control than women, as was the case in the Gesundheit 65+ study. A review on managing hypertension in older adults explained the difficulties associated with hypertension in older women [44]. As well as mentioning some physical changes, such as progressive loss of height or reduced oestrogen production in older age [45, 46], the review also highlights the fact that statins are used less frequently in middle‐aged women than in middle‐aged men, which leads to a higher burden of disease from high BP in older women [47]. This is primarily due to cardiovascular risk being underestimated in women, even in middle age [48].

Treating hypertension in very old adults is complex, as the benefits, e.g., reduced stroke or cardiovascular risk, must be balanced with the risks of excessively low BP, such as frailty and orthostatic hypotension [9, 49, 50]. Antihypertensive medication can increase the risk of frailty, and frailty can also lead to treatment failure [51]. A systematic review of guidelines on the primary prevention of cardiovascular disease shows varying recommendations for the target SBP value in older adults, ranging from < 120 to < 150 mmHg [6]. Accordingly, low BP control in this analysis could indicate that individuals in the sample have higher BP targets than < 140/90 mmHg to prevent negative side effects of low BP. Nevertheless, the control of BP should also be monitored in people over 80, depending on their existing conditions and general health, in order to prevent further cardiovascular complications or adverse events due to low BP.

We used cluster analysis to explore the heterogeneity of older adults with hypertension and to identify patterns that may inform better health care. Cluster analysis can be regarded as an extension of traditional descriptive statistics insofar as it is able to identify subgroups with specific health patterns that remain hidden in an entire population [20, 52]. In our analysis of older adults with hypertension, cluster analysis divided women with hypertension into one group with good awareness, treatment and control of BP but higher burden of multimorbidity and frailty, and a second group with poor BP control but better subjective health and higher life satisfaction. In men, two similar clusters were identified and an additional third cluster with a higher disease burden and more frequent long‐term level of care, but also a high level of hypertension control.

To our knowledge, only a few previous cluster analyses have been conducted in the context of hypertension. However, the study questions and the data differed from those in our study. One study explored a general population sample from 18 to 80 years and identified clusters defined by metabolic and social determinants that had a higher prevalence of hypertension. Their findings underscore the importance of central obesity, dysglycaemia and socioeconomic factors as upstream causes of hypertension [53]. Two studies used cohort data from adults with hypertension to primarily cluster the prognostically relevant combinations of mainly biomarker‐based cardiometabolic risk factors [54, 55]. Both analyses draw attention to individuals with hypertension at increased cardiovascular risk due to a higher metabolic burden.

In contrast, our cluster analysis takes a different and broader approach, including characteristics of hypertension management together with mental health, and typical aspects of old age such as multimorbidity and components of frailty. This highlights that among the oldest, hypertension control is not primarily determined by age or lifestyle factors but rather by treatment status and health system engagement. Individuals with multimorbidity were more likely to achieve BP control. In contrast, less comorbid participants formed a distinct group of undertreated or uncontrolled hypertensives, pointing towards missed opportunities in care provision. Various studies have shown that people with multimorbidity tend to have better BP control [56–58]. This is consistent with our findings. Possible explanations for this phenomenon include more frequent doctor visits, better medication adherence and engagement in the health care system [59].

## 5. Strengths and Limitations

The Gesundheit 65+ study entails representative information on the health status of the old and very old population in Germany. Weighting the sample enabled us to compensate for potential biases caused by varying reparticipation rates across different population groups. The study provides up‐to‐date and unique research data in a sample of people over the age of 80 [21]. Since the Gesundheit 65+ study collected both self‐reported health information and diagnoses via questionnaires and conducted medical examinations, it was possible to carry out a comprehensive assessment of BP and the awareness, treatment and control of hypertension. Furthermore, BP measurements were conducted in participants’ homes, reducing the barriers to participation.

However, there are some limitations that must be considered: (1) Individuals in very poor general health and those residing in nursing homes still may be underrepresented. (2) Survival bias is an issue inherently linked to studies on older adults. As the current life expectancy of older women in Germany is around 4.7 years higher than that of older men [60], the sample is biased in terms of survival probability. To counteract this, sex‐stratified random samples of the population in the age group 80 years and older were then drawn from the local registration registers to include enough men 80 years and older. (3) Further, participants were examined only once. This is in accordance with the prevailing conventions of epidemiological studies and allows the evaluation of BP distribution and trends. (4) The study design allowed for subjective health assessments of proxy participants. This information may be biased, as representatives base their answers on their own perception, affecting 32 people in the sample. An additional analysis, excluding the proxy participants, did not yield any substantially different results in terms of cluster distribution (results not shown). (5) The sample size for the cluster analysis among participants with hypertension is still limited, which results in very small clusters. Nevertheless, we see a major advantage in cluster analysis in this case, as we can comprehensively describe the population over 80 years of age with high BP without predefined assumptions.

## 6. Conclusion

This study provides up‐to‐date and comprehensive data on hypertension prevalence, awareness, treatment and control in the German population over the age of 80. This population of adults aged 80 and over had a high level of awareness of hypertension and its treatment. Yet, there was a sex‐specific difference in terms of lower BP control in women. Cluster analysis contributed to a better understanding of factors linked to better BP management in old women and men. Targeting those identified high‐risk groups of old adults with better general health but higher BP could help improve BP control and, therefore, reduce cardiovascular morbidity and mortality in the older population.

## Author Contributions

Dorothea Möẞnang, Julia Charlotte Büschges and Hannelore Neuhauser designed the study. Dorothea Möẞnang, Julia Charlotte Büschges, Beate Gaertner and Hannelore Neuhauser conceptualised the analysis. Dorothea Möẞnang performed the analysis. Dorothea Möẞnang drafted the manuscript. Dorothea Möẞnang, Julia Charlotte Büschges, Giselle Sarganas, Beate Gaertner, Judith Fuchs and Hannelore Neuhauser contributed to reviewing and editing subsequent drafts and provided critical input to improve the analytical approach, the interpretation of results, the communication of the results and the scope of the findings and conclusions.

## Funding

Gesundheit 65+ was funded by the German Federal Ministry of Health (grant number: ZMVI1‐2518FSB410); in addition, in‐kind funding from the Robert Koch Institute was used as part of the 9‐Point Plan 2b‐31. These analyses were supported by the German Centre for Cardiovascular Research (grant number: MDC2020Z13). Open Access funding enabled and organized by Projekt DEAL.

## Disclosure

This study was presented as poster at the annual conference of the German Society of Epidemiology, September 2025.

## Ethics Statement

Participation in the study was voluntary and could also be declined in parts. For participation in Gesundheit 65+, either the invited person or her/his legal representative gave written consent. The study was approved by the Ethics Committee of the Berlin Chamber of Physicians (German: Berliner Ärztekammer, Eth‐50/19).

## Conflicts of Interest

The authors declare no conflicts of interest.

## Supporting Information

Additional supporting information can be found online in the Supporting Information section.

## Supporting information


**Supporting Information 1** Supporting A: Description of clustering process (Methods). Supporting file A provides a detailed methodological description of the procedure for handling missing values using multiple imputation by chained equations. It also contains an in‐depth description of the factor analysis of mixed data and subsequent PAM clustering process using Euclidean distances.

**Supporting Information 2** Supporting B: Distribution of sociodemographic, anthropometric and health status characteristics of participants according to clusters identified in women and men, selected according to importance for cluster assignment or for the cardiovascular description of the clusters (Results). Supporting file B contains a detailed table summarising the results of the health and health behaviour indicators examined for the five clusters (two for women and three for men), as well as the *p* values used to test for group differences between clusters of one gender.

## Data Availability

The authors state that some access restrictions apply to the data on which the results are based. The data set cannot be made publicly available because the informed consent of the study participants does not cover making the data publicly available. The data set underlying the results is archived at the Research Data Centre of the Robert Koch Institute and can be accessed by researchers upon reasonable request. The data can be accessed on site in the Secure Data Centre of the Research Data Centre of the Robert Koch Institute. Enquiries can be made by e‐mail to fdz@rki.de.
