# Supplementary material for: Blood Pressure and Hypertension Among Adults Aged 80 and Above: Findings From the Population‐Based German Health Survey Gesundheit 65+
Source: Int J Hypertens. 2026 Jul 9;2026:2366213. doi: 10.1155/ijhy/2366213 (PMC13347227; doi:10.1155/ijhy/2366213)
Supplement: Supplementary file 1 — Supporting Information 1 Supporting A: Description of clustering process (Methods). Supporting file A provides a detailed methodological description of the procedure for handling missing values using multiple imputation by chained equations. It also contains an in‐depth description of the factor analysis of mixed data and subsequent PAM clustering process using Euclidean distances. [file IJHY-2026-2366213-s001.docx]

Supplement A: Description of clustering process

For the exploratory identification of clinically meaningful hypertension subgroups, we implemented a sex-stratified clustering pipeline based on the practical Qluster workflow for mixed health data [32]. Analyses were restricted to participants with hypertension according to our study definition and were aged ≥80 years.

Prior to clustering, data quality checks were applied. Infinite values were set to missing. Variables with complete missingness were removed, followed by exclusion of constant or single-level variables (numeric variance=0 or ≤1 factor level), ensuring sufficient feature variability for mixed-data reduction.

The missing values in the data set ranged from 0% to 15%. Exceptions are missing values due to certain filter settings, which can contain up to 45% missing values. Missing values were handled using multiple imputation by chained equations (MICE; m=5, 20 iterations). Imputation models were assigned by measurement scale: predictive mean matching for continuous variables, logistic regression for binary factors, and proportional-odds regression for ordered factors. A full predictor matrix was used to exploit multivariable information. After imputation, one completed dataset was extracted. If any missing values remained, a deterministic fallback imputation was applied (mean for continuous variables; modal category for categorical variables), and constant variables introduced post-imputation were removed. Final datasets contained no missing values.

All subsequent steps were performed separately for men and women. To accommodate mixed variable types and reduce dimensionality, we performed Factor Analysis of Mixed Data (FAMD). The components were then used to create a low-dimensional feature space, which formed the basis for the subsequent cluster analysis. To conduct cluster analyses, we computed Euclidean distances on basis of individual FAMD coordinates. Cluster number was evaluated for k=2–10 via average silhouette width from Partitioning Around Medoids (PAM) solutions, and the optimal k was selected by the maximum silhouette. Final PAM clustering was then fit on the FAMD distance matrix using the chosen k (women: k=2; men: k=3).

Cluster stability was assessed by bootstrap subsampling (B=200, 80% of observations per resample). A fixed FAMD space was defined from the full imputed dataset; each subsample was projected into this space, reclustered with PAM, and compared to the original solution. Cluster-wise Jaccard similarities were computed using best-matching clusters per resample, summarising reproducibility across bootstraps.

For interpretation, cluster assignments were merged back to the original datasets. We described clusters a posteriori using descriptive statistics and between-cluster comparisons, supported by variable distribution summaries. Continuous variables were presented as means and categorical variables as percentages. The differences between the two (three) clusters were analysed using the Wilcoxon Rank-Sum Test (Kruskal–Wallis Test) for continuous variables and the chi-square test for categorical variables. In instances where the anticipated cell frequencies were deemed to be inadequate, the exact Fisher test was employed. A p-value of less than 0.05 was considered to be significant.
