# Supplementary material for: Blood Pressure and Hypertension Among Adults Aged 80 and Above: Findings From the Population‐Based German Health Survey Gesundheit 65+
Source: Int J Hypertens. 2026 Jul 9;2026:2366213. doi: 10.1155/ijhy/2366213 (PMC13347227; doi:10.1155/ijhy/2366213)
Supplement: Supplementary file 2 — Supporting Information 2 Supporting B: Distribution of sociodemographic, anthropometric and health status characteristics of participants according to clusters identified in women and men, selected according to importance for cluster assignment or for the cardiovascular description of the clusters (Results). Supporting file B contains a detailed table summarising the results of the health and health behaviour indicators examined for the five clusters (two for women and three for men), as well as the p values used to test for group differences between clusters of one gender. [file IJHY-2026-2366213-s002.docx]

*Supplement B: Distribution of sociodemographic, anthropometric, and health status characteristics of participants according to clusters identified in women and men, selected according to importance for cluster assignment or for the cardiovascular description of the clusters.*

|  | Women | | | Men | | | |
| --- | --- | --- | --- | --- | --- | --- | --- |
|  | Cluster 1 N = 241 | Cluster 2 N = 71 | p-value | Cluster 1  N = 111 | Cluster 2  N = 96 | Cluster 3  N = 63 | p-value |
| Age (Mean) | 84.8 | 84.5 | 0.230 | 83.8 | 84.4 | 85.4 | 0.020 |
| **Blood pressure** | | | | | | | |
| SBP (Mean) | 135.6 | 154.6 | <0.001 | 122.2 | 145.5 | 126.7 | <0.001 |
| DBP (Mean) | 78.3 | 90.1 | <0.001 | 73.8 | 88.3 | 75.7 | <0.001 |
| **Hypertension treatment cascade** | | | | | | | |
| Awareness (%) | 98.0 | 31.0 | <0.001 | 100.0 | 55.0 | 100.0 | <0.001 |
| Treatment (%) | 97.0 | 23.0 | <0.001 | 100.0 | 52.0 | 94.0 | <0.001 |
| Control (%) | 61.0 | 2.9 | <0.001 | 94.0 | 16.0 | 70.0 | <0.001 |
| **Antihypertensive medication** | | | | | | | |
| Diuretics (%) | 57.0 | 7.0 | <0.001 | 53.0 | 20.0 | 71.0 | <0.001 |
| Betablocker (%) | 61.0 | 14.0 | <0.001 | 60.0 | 27.0 | 62.0 | <0.001 |
| Calcium channel blockers (%) | 51.0 | 5.6 | <0.001 | 58.0 | 15.0 | 37.0 | <0.001 |
| ACE inhibitors (%) | 31.0 | 13.0 | 0.002 | 33.0 | 31.0 | 37.0 | 0.789 |
| Angiotensin receptor blockers (%) | 51.0 | 17.0 | <0.001 | 55.0 | 23.0 | 38.0 | <0.001 |
| Other anti-hypertensive drugs (%) | 4.6 | 0.0 | 0.075 | 4.5 | 1.0 | 6.3 | 0.170 |
| Antihypertensive polytherapy (%) | 85.0 | 31.0 | <0.001 | 94.0 | 52.0 | 90.0 | <0.001 |
| **Physical health** | | | | | | | |
| Obesity (%) | 30.0 | 11.0 | 0.001 | 17.0 | 19.0 | 33.0 | 0.057 |
| (Very) good subjective health (%) | 30.0 | 59.0 | <0.001 | 57.0 | 70.0 | 11.0 | <0.001 |
| Multimorbidity (%) | 90.0 | 55.0 | <0.001 | 86.0 | 62.0 | 94.0 | <0.001 |
| **Frailty components** | | | | | | | |
| Low energy (%) | 26.0 | 9.1 | 0.003 | 7.7 | 1.1 | 63.0 | <0.001 |
| Unintentional weight loss (%) | 6.1 | 0.0 | 0.045 | 2.8 | 1.1 | 13.0 | 0.003 |
| Low physical activity (%) | 40.0 | 23.0 | 0.010 | 17.0 | 19.0 | 70.0 | <0.001 |
| Walking difficulties (%) | 40.0 | 13.0 | <0.001 | 7.6 | 3.4 | 81.0 | <0.001 |
| Low grip strength (%) | 43.0 | 31.0 | 0.076 | 42.0 | 38.0 | 73.0 | <0.001 |
| **Mental health** | | | | | | | |
| Depressive symptoms (%) | 19.0 | 14.0 | 0.327 | 5.7 | 2.3 | 51.0 | <0.001 |
| Subjective memory impairment (%) | 40.0% | 42.0% | 0.793 | 30.0% | 27.0% | 53.0% | 0.003 |
| Life satisfaction (%) | 61.0 | 85.0 | <0.001 | 79.0 | 88.0 | 36.0 | <0.001 |
| **Sociodemographic characteristics** | | | | | | | |
| Care level (%) | 43.0 | 17.0 | <0.001 | 12.0 | 6.7 | 69.0 | <0.001 |
